# Genetic Diversity and Population Structure of *Siberian apricot* (*Prunus sibirica* L.) in China

**DOI:** 10.3390/ijms15010377

**Published:** 2013-12-31

**Authors:** Ming Li, Zhong Zhao, Xingjun Miao, Jingjing Zhou

**Affiliations:** State Key Laboratory of Soil Erosion and Dryland Farming on the Loess Plateau, Northwest A&F University, Yangling 712100, China; E-Mails: limingly@126.com (M.L.); miaoxingjun@nwsuaf.edu.cn (X.M.); hupodingxiangyu@163.com (J.Z.)

**Keywords:** genetic diversity, population structure, *Prunus sibirica*, *Siberian apricot*

## Abstract

The genetic diversity and population genetic structure of 252 accessions from 21 *Prunus sibirica* L. populations were investigated using 10 ISSR, SSR, and SRAP markers. The results suggest that the entire population has a relatively high level of genetic diversity, with populations HR and MY showing very high diversity. A low level of inter-population genetic differentiation and a high level of intra-population genetic differentiation was found, which is supported by a moderate level of gene flow, and largely attributable to the cross-pollination and self-incompatibility reproductive system. A STRUCTURE (model-based program) analysis revealed that the 21 populations can be divided into two main groups, mainly based on geographic differences and genetic exchanges. The entire wild *Siberia apricot* population in China could be divided into two subgroups, including 107 accessions in subgroup (SG) 1 and 147 accessions in SG 2. A Mantel test revealed a significant positive correlation between genetic and geographic distance matrices, and there was a very significant positive correlation among three marker datasets. Overall, we recommend a combination of conservation measures, with *ex situ* and *in situ* conservation that includes the construction of a core germplasm repository and the implement of *in situ* conservation for populations HR, MY, and ZY.

## 1. Introduction

Evaluation of the level and structure of genetic diversity in natural populations of a species is important for plant breeding and genetic resource conservation programs [1]. Indeed, wild species play crucial roles in breeding programs because of their wide variability in terms of phenological, morphological, abiotic, biotic, and quality traits [2]. However, it has been widely reported that a large amount of genetic diversity has been lost in major crops due to drift and selection in comparison to the wild forms, thereby reducing the potential for crop improvement in modern agricultural systems [3]. Genetic variation must exist to maintain natural populations as evolutionarily viable units capable of adapting to changing environmental conditions in the long term [4]. Thus, a genetic resource management strategy should involve an investigation of the genetic diversity and the extent of genetic differentiation within and between populations and an understanding of the processes that maintain these variations.

In this work, we present a case study of *Siberian apricot* (*Prunus sibirica* L.) in China. *Siberian apricot*, a member of the genus *Prunus* belonging to the family Rosaceae, is an important ecological and economic tree species endemic to Asia. *P. sibirica* is distributed in regions of eastern Siberia, including the maritime territory of Russia, eastern regions of Mongolia, and northern regions of China [5]. In China, *Siberian apricot* is distributed throughout a total area of approximately 2 million hectare, with an annual seed production of nearly 1 million tons. It is able to grow in environments with a low temperature, strong wind, low rainfall, and poor soil. Considering its ecological benefits, such as water and soil conservation, windbreak, sand fixation, and environmental protection and greening, *Siberian apricot* is often used for afforestation in north China [6]. The seeds of *P. sibirica* also have important economic values: the seed shell can be pyrolyzed to activated carbon and pyroligneous liquor [7]; the seed kernel can be processed for protein drinks [8]; the seed kernel oil can be used for edible oils, cosmetics, biodiesel, and in the prevention of cardiovascular diseases and lowering of plasma cholesterol levels [9,10]. Traditional Chinese medicine uses the seed kernels in different preparations for treating asthma, coughs, and infant virus pneumonia [11].

Furthermore, *Siberian apricot* is an important breeding resource for apricot production. According to molecular studies, many apricot cultivars grown for fruit, particularly in China, belong to the species *P. armeniaca* obtained through introgression with *P. sibirica* [12]. For adaptation to the arid and cold environment in China and Russia, some cultivated apricots have been bred from *P. sibirica* or obtained by grafting with *P. sibirica* [13]. In addition, Chinese apricots and/or *P. sibirica* have been important for introgressing resistance to Plum pox virus (PPV) in North American germplasm [12]. The increased attention to the industrial value of *Siberian apricot* in recent years has highlighted the often low fruit set and tree productivity due to late-season frosts and a lack of variety breeding, with an insufficient fruit supply for industrial development. Therefore, the use of diverse wild resources to broaden the genetic base and increase the potential for the ecological adaptation of *Siberian apricot* cultivars allows important objectives to be pursued in breeding. Unfortunately, due to human interference, the area originally covered by wild resources has been greatly reduced, with the remaining area mostly found on mountains and cliffs. Thus, the genetic diversity and population structure of *Siberian apricot* in China should be immediately investigated for resource conservation and breeding.

DNA markers allow the direct assessment of relatedness at the DNA level by estimating the proportion of alleles in individuals and populations. Different molecular markers can be used for genomic analysis, and simple sequence repeats (SSR), inter-simple sequence repeats (ISSR), and sequence-related amplified polymorphism (SRAP) markers have been recognized as useful molecular markers for diversity studies, population genetic analysis, and other purposes in various species [14–16]. SSR markers have been well applied in apricot genetics due to the advantages of abundance in the apricot genome, co-dominance, and a high polymorphism rate [17–20]. ISSR markers are based on amplification of the specific regions between two SSR sequences, with the advantages of deep genome coverage, high effectiveness (time-effective and cost-effective manner), and wide use in apricot [21–23]. Recently used in apricot, SRAP markers target open reading frames (ORFs), combining simplicity, reliability, a moderate throughput ratio, and the disclosure of co-dominant markers [24,25]. However, as there are few different and combined analyses of these three markers, it is important for researchers to compare these markers to identify the approaches that best address the research concerns.

To our knowledge, there is no research to date focusing on the genetic diversity and population structure of *Siberian apricot* in China. Thus, the objectives of this study were to (i) characterize the genetic diversity and genetic differentiation of 21 *Siberian apricot* populations using SSR, ISSR, and SRAP markers; (ii) characterize the genetic structure in the populations; (iii) assess the correspondence among the three markers; and (iv) offer an effective conservation strategy for wild *Siberian apricot*.

## 2. Results and Discussion

### 2.1. Genetic Diversity Analysis

Ten respective primers for SSR, ISSR, and SRAP markers amplified 45, 103, and 120 putative genetic loci, with a total of 268 loci detected. For the SSR markers, we detected 4.50 observed number of alleles and 3.77 effective number of alleles; for the ISSR and SRAP markers, 91.3% and 82.5% bands, respectively, were polymorphic (Tables S1 and S2). Nei’s gene diversity (*h*), Shannon’s information index (*I*), and the percentage of polymorphic bands (*PPB*) were used to assess the genetic diversity (Table 1). The ISSR markers revealed the highest genetic diversity in population HC (*h* = 0.208, *I* = 0.310, *PPB* = 59.2%), followed by populations HR and AS, with a high level of diversity at the species level (*h* = 0.248, *I* = 0.387, *PPB* = 91.3%). According to the SRAP markers, population HR had the highest genetic diversity (*h* = 0.213, *I* = 0.326, *PPB* = 69.2), followed by populations ZY and MY, with a weaker level of diversity at the species level (*h* = 0.218, *I* = 0.344, *PPB* = 82.5%) than the ISSR markers. Based on the SSR markers, the highest genetic diversity was present in population AS (*h* = 1.231, *I* = 0.662), followed by populations MY and HC, with a high level of diversity at the species level (*h* = 1.639, *I* = 0.782). Populations KK and LX showed the lowest genetic diversity index using these three markers.

Compared to previous research in apricot species, the *Siberia apricot* in China showed a relatively high level of genetic diversity, higher than cultivated apricot (*P. armeniaca*) in Turkey revealed by ISSR markers (*PPB* = 88%) but lower than wild apricot (*P. armeniaca*) in the Ili Valley (*PPB* = 94.84%) [23,26]. The expected heterozygosity (0.713) revealed by the SSR markers was also higher than the apricot accessions (*P. armeniaca*) in the Maghreb region (0.593), but similar to the core collection apricot germplasm in China (0.731) [27,28]. As the same wild apricot resource, *Siberia apricot* has a slightly weaker genetic diversity index than wild apricot in the Ili Valley (*PPB* = 91.3% *vs.* 94.84%) but a larger population size and distribution area. As wild apricot in the Ili Valley has been accepted as the oldest and most diverse natural population, we suggest that the natural *Siberia apricot* populations in China retain a relatively high level of genetic diversity [29].

The HR and MY populations showed much higher diversity parameters and are close in geographical distance. The two populations are located at the western end of the Yanshan Mountains, a traditional *Siberia apricot* growing region. Because of their economic value, the *Siberia apricot* seeds are widely collected by villagers in north China, and most populations will be affected by human disturbance [10,30]. The areas in which the HR and MY population are located have an advanced economy, with most of the apricot resources being distributed on rocky cliff and humans rarely collecting the seeds. We suggest that the reduced impact of human disturbance is the reason for the higher diversity in populations HR and MY. With the exception of population ZD, the populations (ZY, LY, AS, HC, and HX) in northwest China all showed higher diversity parameters than the mean. This region has a warmer climate for *Siberia apricot* growth and overlaps with the *P. armeniaca* distribution. In recent years, apricots grown for the kernels have been cultivated in this region, including hybrid varieties of *P. armeniaca* and *P. sibirica* [31]. We believe that a genetic exchange with *P. armeniaca* occurred with *P. sibirica* in this region, affecting the genetic diversity of *P. sibirica*.

### 2.2. Genetic Differentiation Analysis

Assuming Hardy-Weinberg equilibrium, the genetic differentiation coefficient was calculated from the allele frequencies estimated according to the square root method using POPGENE. As revealed by the ISSR markers, the relative magnitude of genetic differentiation among the populations (*G*_ST_) was 0.18. An AMOVA analysis showed that 25.01% of the total gene diversity was found among the natural populations, whereas the remaining 74.99% of the total variation occurred within the populations (Table 2). The SRAP markers showed that the *G*_ST_ among the populations was 0.28, indicating that 76.16% of the total variance occurred within the populations. According to the SSR markers, the genetic differentiation among the populations (*F*_ST_) was 0.15, indicating that 83.35% of the total variance occurred within the populations. The three markers all indicated a low level of inter-population genetic differentiation and high level of intra-population genetic differentiation in wild *Siberia apricot*. This result was further confirmed by the moderate level of gene flow (*N*_m_) between the populations (1.58 for ISSR, 1.28 for SRAP, 1.37 for SSR).

Based on the values *Fst* (0.15) and *Gst* (0.18 and 0.28), a large amount of genetic variation in *Siberia apricot* was found within the populations, equivalent to the differentiation among the natural populations observed in wild apricot of the Ili valley (*Fst* = 0.137; *Gst* = 0.164) but lower than the North Africa apricot populations (*Fst* = 0.04) according to SSR markers [27,29]. Due to the cross-pollination reproductive system and self-incompatibility, we determined that *Siberia apricot* in China is an outcrossing species. A value of *Nm* > 1 indicates no significant genetic differentiation among populations [32]. In the present study, the gene flow was moderate and revealed a high level of genetic diversity maintained within the populations that was not susceptible to genetic drift. The mode of pollen and seed dispersal, which determines gene flow among populations, may partly account for this moderate differentiation. Although the pollen of *Siberia apricot* can be spread over a long distance by the combination of insects and wind, the large distribution area and largely discontinuous distribution negate this possibility. As a long-lived perennial woody plant, *P. sibirica* is widely distributed in regions of eastern Siberia, with large population sizes. To adapt to ecologically diverse habitats, it has probably accumulated considerable genetic variation within species. The populations in China may be characterized by shared ancestral polymorphisms, and have maintained large effective population sizes so that the shared variation has not been lost by drift. Considering the partly overlapping distribution with *P. armeniaca* and *P. mandshurica*, the variation found among the *P. sibirica* populations was possibly due to drift or crossing with other *Prunus* species. In addition, *P. sibirica* uses an animal-ingested seed dispersal system; its efficiency largely depends on the migration habits and activity of animals. Obligate fruit-eaters that feed mainly or solely on the fruit of *P. sibirica* during the maturation period, when fruit-ripening asynchrony occurs, may confine their activities within the areas where ripe fruits are available. As a result, the seed exchange among *P. sibirica* populations will be minimal. In addition, factors such as the low seed germination rate and breeding system may also partly contribute to the present population genetic differentiation in *P. sibirica*.

### 2.3. Population Structure and Cluster Analysis

To further elucidate the relationships among the populations, Nei’s unbiased measure of genetic distance was applied to calculate the genetic distances between them (Tables S3–S5); based on the results, a Cluster analysis of the distance matrices based on an UPGMA algorithm was used to generate a dendrogram. The population structure was analyzed using a Bayesian approach on 252 accessions implemented in the STRUCTURE software (version 2.3.4; Pritchard J.K., Stanford, CA, USA. http://pritchardlab.stanford.edu/structure.html) [33]. Following the method of Evanno [34], the Δ*K* values were plotted against the *K* numbers of the sub-groups. The maximum Δ*K* occurred at *K* = 2 for the three markers (Figure S1). We divided the accessions into different sub-groups considering membership probabilities of ≥0.50, and we incorporated the populations to two main groups according to the sub-group of the major accessions.

**Figure 1.**
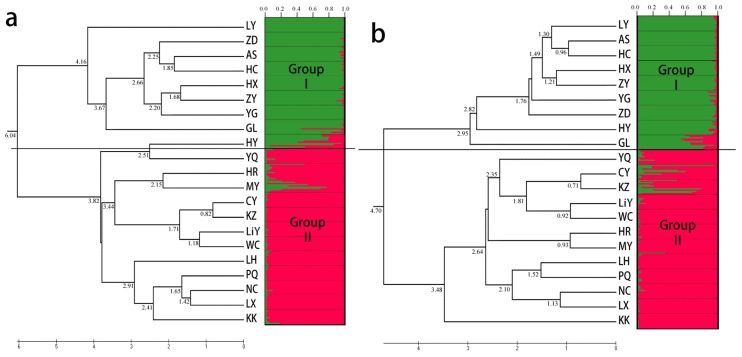

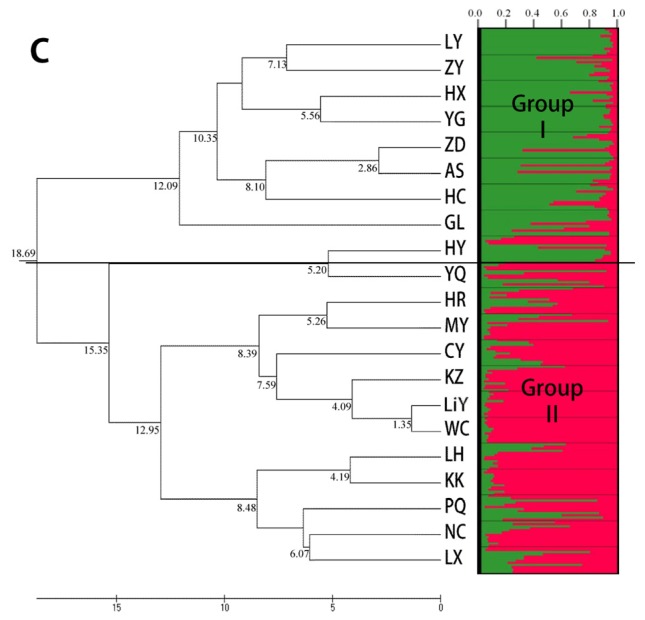
UPGMA dendrogram for the wild *Siberia apricot* populations based on Nei’s genetic distance [40], as revealed using (**a**) ISSR markers; (**b**) SRAP markers; and (**c**) SSR markers. The distance coefficients between populations obtained by Cluster analyses are marked on branches and the *x*-axis.

**Figure 2.**
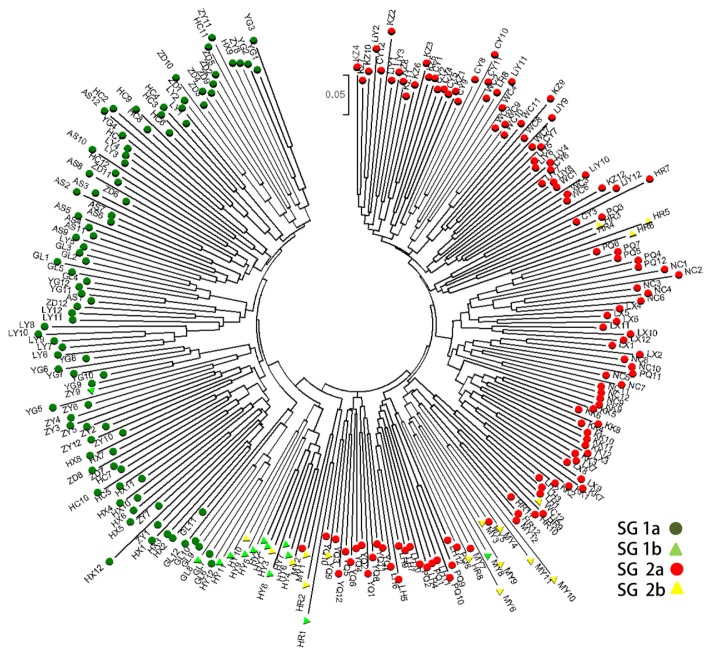
Neighbor-joining trees of 252 wild *Siberia apricot* accessions.

Considering the ISSR markers, the dendrogram divided the 21 populations into two main clusters, a large cluster with 13 populations and a small with 8 populations (Figure 1a). The STRUCTURE analysis indicated that the entire population could be divided into two groups: group I consisted of 9 populations, LY, ZD, AS, HC, HX, ZY, YG, GL, and HY; the other populations were clustered into group II (Figure 2a). Similar results were obtained with the STRUCTURE analysis and Cluster analysis, except for population HY. Five accessions in population HY had a similar genetic structure as group II. The Cluster analysis based on the SRAP data generated a unique dendrogram that divided the 21 populations into two main clusters, similar to the subgroups of the STRUCTURE analysis (Figure 1b). Group I consisted of populations LY, ZD, AS, HC, HX, ZY, YG, GL, and HY, with the other populations clustered into group II, similar to the results using the ISSR markers. The dendrogram of the Cluster analysis based on the SSR data divided the 21 populations into two main clusters, in agreement with the dendrogram based on the ISSR data (Figure 1c). When considering *K* = 2, the populations were split into two groups: populations LY, ZD, AS, HC, HX, ZY, YG, GL, and HY in group I and the remaining populations in group II.

The population genetic structure reflects interactions among species with regard to their long-term evolutionary history, mutation and recombination, genetic drift, reproductive system, gene flow, and natural selection [35,36]. Thus, an understanding of the level and structure of the genetic diversity of a crop is a prerequisite for the conservation and efficient use of the germplasm available for breeding [2]. In the present study, we analyzed the information obtained using three markers, and all of them indicated two groups according to dendrogram and STRUCTURE analyses, though a slight difference was found regarding population HY. Group I included populations LY, ZD, AS, HC, HX, ZY, YG, GL, and HY, and group II included populations YQ, HR, MY, CY, KZ, LiY, WC, LH, PQ, NC, LX and KK. It is noteworthy that the two groups appear to be divided by geographic distribution, with group I distributed in west of 115 °E and group II distributed in east of 115 °E. The dendrogram topology was generally consistent with the geographic distribution of these populations, indicating a possible correlation, and the Mantel test of correlation between the genetic and geographic distance matrices revealed a significant positive correlation (ISSR, *r* = 0.7379; SRAP, *r* = 0.6160; SSR, *r* = 0.5490; *p* ≤ 0.001). Most of the area of group I is located in the warm-temperate zone in northwest China, and group II is located in the mid-temperate zone in northeast and north China, and there is a large difference in light, temperature, and other climate conditions in these two regions [37]. For example, late-season frosts are a serious issue for apricot growth, and the duration and timing of frosts are different in these two regions, thus affecting the survival and blossoming time of *Siberia apricot* [38]. Plant growth and development are sensitive to climate [39]. In our field investigations, we found that the height of mature *Siberia apricot* trees was approximately 3–6 m in group I and 1.5–5 m in group II; thus, we hypothesized that environmental differences would affected the genetic structure of *P. sibirica* populations in the long term. In northern China, the distribution of *P. sibirica* overlaps with wild *P. armeniaca* and *P. mandshurica* [11]; The *P. mandshurica* is completely distributed in northeastern China, the region in which most of the group II populations are found. We hypothesized that genetic exchange of group I and group II with different species of *Prunus* would also affect the difference in genetic structure between the groups. In general, geographic difference and genetic exchange should be mainly responsible for the current genetic structure of populations.

### 2.4. Comparison of ISSR, SSR, and SRAP Markers

The three markers resulted in similar dendrogram and sub-group division results, revealing a possible correlation and uniformity among them. To obtain a more robust comparison, a Mantel matrix correspondence test was used in matrices of the genetic distance values of 252 accessions generated using the three markers. The test revealed a very significant positive correlation among them (ISSR with SRAP *r* = 0.766, ISSR with SSR *r* = 0.694, SRAP with SSR *r* = 0.631, *p* < 0.01). The results showed some differences with a report by Budak using ISSR, SSR, RAPD, and SRAP markers in buffalo grass (*Buchloe dactyloides*) [41], which indicated a significant positive correlation between ISSR and SSR (*r* = 0.66, *p* < 0.01), but non-significant correlation between SRAP and the other markers.

Despite the great and similar discriminating power of each marker system used, there were some differences detected. For instance, the number of total polymorphic and discriminant fragments was higher for the ISSR markers (94 polymorphic fragments), showing a higher capacity to reveal polymorphisms than SRAP. Previous research supports a higher capacity of ISSRs to reveal polymorphisms and to demonstrate a great potential to determine the intra- and inter-genomic diversity as compared to other arbitrary primers [42]. In this study, the SSR markers indicated a higher genetic structure difference among the populations. The SRAP markers showed the highest number of polymorphic and discriminating fragments (99 polymorphic fragments), demonstrating uniformity in the Cluster analysis and STRUCTURE analysis. In addition, as ISSRs and SRAPs are the dominant marker systems, the ancestral dissection of polyploids might be difficult for the comparison of SSR markers. Hence, the co-dominant nature of SSR markers would make them the marker of choice for segregation studies and genome mapping in apricot.

### 2.5. Combined Analysis

Few studies have compared the results obtained from individual *vs.* combined molecular marker datasets for the purpose of genetic diversity analysis [41,43]. In this study, the complete accession datasets were combined to reveal the true genetic structure in wild *Siberia apricot* populations. The SSR data were converted into a dominance data matrix to generate a dendrogram with combined ISSR and SRAP data. The software NTSYS 2.11 was applied to construct the neighbor-joining tree on the basis of the Jaccard similarity coefficient [44]. Following the method of Evanno [34], the obvious optimum Δ*K* occurred at *K* = 2 (Figure S1), which indicated that the entire population could be divided into two subgroups (*i.e.*, SG 1 and SG 2) (Figure 2). With membership probabilities of ≥0.50, 107 wild *Siberia apricot* accessions were assigned to SG 1, and the other 147 accessions were assigned to SG 2. SG 1 and SG 2 could be further divided into four groups, with 94 and 128 accessions assigned to SG 1a and SG 2a (membership probabilities of ≥0.80), and 13 and 17 accessions assigned to SG 1b and SG 2b (0.50≤ membership probabilities of <0.80) [45]. SG 1b and SG 2b appear to have much stranger hybrids and a complicated genetic background. Furthermore, the neighbor-joining (NJ) tree showed 3 branches within the entire population, which was weakly consistent with the STRUCTURE analysis based on membership assignment (Figure 2). The PCoA analysis by NTSYS 2.11 graphically showed two distinct clusters for the entire population (Figure S2), a result that was highly related to the known germplasm information and the STRUCTURE subgroups.

The Cluster analysis or STRUCTURE analysis revealed that most accessions from the same population aggregated together. In population HY, 9 accessions were in placed into SG 1b, with the other 3 accessions being placed into SG 2b. The STRUCTURE analysis of some graft- and seed-propagated apricots in North African showing *K* = 2 and *K* = 4 were considered to best depict the genetic structure, reflecting a slight difference with our results [46]. Research on cultivated apricot in Spain revealed the best *K* value as 5 or 7, rather different from our results [47]. Differences in the species class, population size and distribution, and genetic background might lead to such results.

### 2.6. Conservation Considerations

The main objective in any plant genetic resource conservation program should be to maintain the highest possible level of genetic variability [48]. According to the results of our field survey, anthropogenic activities, such as fruit picking, seed collection, deforestation, and grazing, have undoubtedly influenced the natural habitats and reduced the area of wild *Siberia apricot* distribution. Seed collection might represent the highest threat for wild *Siberia apricot* at present and in the future due to the development of the *Siberia apricot* seed industry [5,10,28]. These activities will inevitably affect population regeneration, which would hinder resource conservation and economic development. This dilemma should be resolved through conservation measures.

To preserve the valuable wild *Siberia apricot* genetic resources and considering the large distribution area and dispersal situation, we recommend a combination of conservation measures that include *ex situ* and *in situ* conservation. Firstly, we recommend the construction of a core germplasm repository and the collection of germplasm resources in greater breadth and depth. This will allow seeds to be collected and exchanged between populations, increasing the genetic diversity in each area and conserving the scarce germplasm resources through natural regeneration. In fact, more attention should be given to *ex situ* efforts because it would be difficult to implement *in situ* conservation for all the populations due to the large distribution area and dispersal. Secondly, *in situ* conservation should be implemented immediately. Given their high level of genetic diversity and desirable growth patterns in their original habitat, populations HR and MY should be assigned a high priority. Additionally, the genetic diversity of the two subgroups should be protected, and a priority conservation measure should be instituted for population ZY. These measures should include the establishment of nature reserves and forest reservation and the banning of grazing. Thirdly, seed collection activity using wild resources should be controlled, and the development of cultivated *Siberia apricot* resources should be encouraged. For the sake of industrial development and local economies, the commercial planting of *Siberia apricot* should be established in suitable areas.

## 3. Experimental Section

### 3.1. Plant Materials

**Figure 3.**
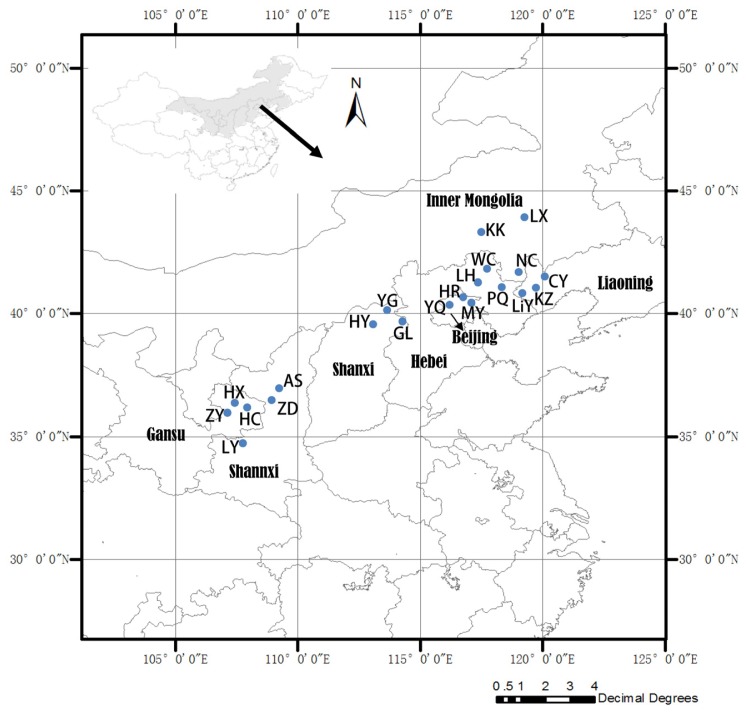
Geographic distribution of the 21 *P. sibirica* populations sampled in this study.

A total of 252 trees (12 trees per provenance) were collected from north China, a region with a continuous distribution of *Siberian apricot* in July and August 2012 (Figure 3). The collections covered a broad environmental span, with the longitudes ranging from N 34°35′ (LY, Shaanxi Province, China) to 44°01′ (LX, Inner Mongolia, China), latitudes ranging from 107°12′ (ZY, Gansu Province, China) to 120°03′ (CY, Liaoning Province, China), and elevations spanning from 228 to 1506 m. Every population consisted of 12 individuals, and more than 50 m separated each pair of individuals. The locations of the 21 populations are listed in Table 3. The seeds of every individual were collected, and the seed testas were used for DNA extraction.

### 3.2. DNA Extraction

The maternal tissues forming the testa were separated from the rest of the seed (embryo), and genomic DNA was extracted using the modified DNA extraction protocol of Martin and Li [26,47]. The DNA concentration was measured using an Epoch™ microplate spectrophotometer (BioTek, Winooski, VT, USA) and was diluted to a working concentration of 30 ng/μL.

### 3.3. DNA Amplification

The extracted apricot genomic DNA was separately amplified by PCR using 10 ISSR, SSR, and SRAP primers; the primers sequences and sources are listed in Tables S1 and S2 [16,24,49–54]. The amplifications were performed using a thermal gradient cycler (Applied Biosystems, Foster, CA, USA) with a total volume of 15 μL containing 30 ng genomic DNA, 0.25 mM primer, 3 mM MgCl_2_, 0.4 mM dNTPs, and 1 U Taq DNA Polymerase. The ISSR reactions were performed with an initial step of 3 min at 95 °C, followed by 35 cycles of denaturation at 94 °C for 60 s, annealing for 60 s, and extension at 72 °C for 120 s; a final extension was performed for 5 min at 72 °C. The SRAP cycling parameters included 3 min at 95 °C, 5 cycles of three steps of denaturing at 94 °C for 60 s, annealing at 35 °C for 60 s and extension at 72 °C for 60 s. In the following 35 cycles, the annealing temperature was increased to 50 °C, and one cycle of 5 min at 72 °C was used for extension. The ISSR and SRAP amplification products were analyzed by 1.8% agarose gel electrophoresis in 1× TBE buffer and stained with ethidium bromide; the products were photographed using a Gel Documentation System (Bio-Rad, Hercules, CA, USA). The amplifications for the SSR analysis were performed with an initial step of 3 min at 95 °C, followed by 35 cycles of denaturation at 94 °C for 30 s, annealing at 51 to 57 °C (depending on the primer) for 30 s, and extension at 72 °C for 60 s; a final extension was performed for 5 min at 72 °C. The PCR products were separated by electrophoresis through a 6% denaturing polyacrylamide gel, and the fragments were visualized by silver staining. The genotypes were assessed by eye on the fluorescent plate.

### 3.4. Data Analysis

The ISSR and SRAP amplification fragments were scored according to a binary matrix where 0 and 1 coded for the absence and presence of a band, respectively. For each SSR locus, the allelic composition and number of total alleles were determined for each accession.

The genetic diversity was assessed using the program POPGENE 1.32 [55], as measured by the percentage of polymorphic bands (*PPB*), Nei’s gene diversity (*h*), and Shannon’s information index (*I*). The coefficient of gene differentiation (*G*_ST_, *F*_ST_) and gene flow (*N*_m_) between the populations were also calculated using this program [35,40,56]. The AMOVA means obtained in Arlequin 3.11 were also used to calculate the genetic differentiation among the populations [57]. Mantel 2.0 was used to determine the correlation between the inter-population genetic distance and geographic distance matrices [58]. The SSR genotype banding patterns were converted into a “1” (present) and “0” (absence) matrix, and the tree topologies were constructed based on the neighbor-joining method using NTSYS 2.11 and MEGA 4 [44,59,60].

Genetic relationships among individuals were assessed by a multivariate principal component analysis (PCoA) performed with NTSYS 2.11 (Applied Biostatistics, Setauket, NY, USA) to identify the number of groups based on eigen vectors. Population structure was determined using the model-based program, STRUCTURE [33]. To identify the number of populations (*K*) capturing the major structure in the data, we used a burn-in period of 50,000 Markov Chain Monte Carlo iterations and 100,000 runs, with an admixture model following Hardy-Weinberg equilibrium, and correlated allele frequencies and independent loci for each run. Seven independent runs were performed for each simulated value of *K*, ranging from 2 to 20. The true *K* value was determined using both an estimate of the posterior probability of the data for a given *K* (as proposed by Pritchard *et al.*) and the Evanno Δ*K* [33,34].

## 4. Conclusions

In conclusion, our data initially confirm that all sets of ISSR, SSR, and SRAP markers provide an accurate picture of the population structure within wild *Siberia apricot* collections, information that is of critical importance for the design of genetic diversity and structure analyses. First, the results suggest that wild *Siberia apricot* in China has a relatively high level of genetic diversity; the populations HR and MY show very high diversity parameters, which was attributed to their being less affected by human disturbance. A low level of inter-population genetic differentiation and a high level of intra-population genetic differentiation were found, which was supported by a moderate level of gene flow. We believe that the predominant genetic variation found in wild *Siberia apricot* is attributable to the differences within populations, and is caused by the cross-pollination and self-incompatibility of this plant. Second, the STRUCTURE analysis indicated that the 21 populations can be divided into two main groups. The LY, ZD, AS, HC, HX, ZY, YG, GL, and HY populations were assigned to group I, and the other populations were assigned to group II. The geographic differences and genetic exchange should be mainly responsible for the observed genetic structure of the populations. The Mantel matrix correspondence test revealed a very significantly positive correlation among the datasets of the three markers. Third, the entire wild *Siberia apricot* population in Chian could be divided into two subgroups, with 107 accessions in SG 1 and 147 accessions in SG 2. Furthermore, the Mantel test revealed a significant positive correlation between the genetic and geographic distance matrices. Lastly, we recommend a combination of conservation measures, with *ex situ* and *in situ* conservation, such as the construction of a core germplasm repository, the collection of germplasm resources in greater breadth and depth, and the implementation of *in situ* conservation in populations HR, MY, and ZY. The information obtained from this collection of genotypes will be helpful for the development of good varieties for breeding programs and the conservation of the genetic resources of wild *Siberia apricot* in China.

## Supplementary Information

**Table S1.** SSR and SRAP primers that produced repeatable polymorphic amplification patterns for the genotypes studied.

Primer	Annealing temp (°C)	Total bands	Polymorphism bands	Sequence (5′–3′)	Reference
BC807	50	10	10	(AG) _8_T	UBC Primer Set #9 [ 61]
BC818	52	9	9	(CA) _8_G	UBC Primer Set #9 [ 61]
BC827	51	10	10	(AC) _8_G	UBC Primer Set #9 [ 61]
BC835	54	15	15	(AG) _8_YC	UBC Primer Set #9 [ 61]
BC843	50	8	7	(CT) _8_GA	UBC Primer Set #9 [ 61]
BC847	54	13	13	(CA) _8_RC	UBC Primer Set #9 [ 61]
BC868	48	8	6	(GAA) _6_	UBC Primer Set #9 [ 61]
BC873	49	11	9	(GACA) _4_	UBC Primer Set #9 [ 61]
BC880	48	8	7	(GGAGA) _3_	UBC Primer Set #9 [ 61]
BC888	50	11	8	BDB(CA) _7_	UBC Primer Set #9 [ 61]
Me1/Em1	50	15	14	TGAGTCCAAACCGGAGC/GACTGCGTACGAATTTGC	G. Li *et al.* [ 16]
Me1/Em4	50	9	6	TGAGTCCAAACCGGAGC/GACTGCGTACGAATTGAG	G. Li *et al.* [ 16]
Me2/Em1	50	9	6	TGAGTCCAAACCGGACC/GACTGCGTACGAATTTGC	G. Li *et al.* [ 16]
Me2/Em3	50	12	11	TGAGTCCAAACCGGACC/GACTGCGTACGAATTAAC	G. Li *et al.* [ 16]
Me2/Em9	50	10	6	TGAGTCCAAACCGGACC/GACTGCGTACGAATTATT	PF. Ai *et al.* [ 24]
Me4/Em7	50	11	8	TGAGTCCAAACCGGTCC/GACTGCGTACGAATTGCA	PF. Ai *et al.* [ 24]
Me5/Em2	50	9	8	TGAGTCCAAACCGGTGC/GACTGCGTACGAATTTGA	G. Li *et al.* [ 16]
Me8/Em9	50	9	8	TGAGTCCAAACCGGAAG/GACTGCGTACGAATTATT	PF. Ai *et al.* [ 24]
Me1/Em6	50	19	17	TGAGTCCAAACCGGAGC/GACTGCGTACGAATTCTT	PF. Ai *et al.* [ 24]
Me8/Em8	50	17	15	TGAGTCCAAACCGGAAG/GACTGCGTACGAATTGCC	PF. Ai *et al.* [ 24]

**Table S2.** List of SSR primers and Genetic diversity parameters for the genotypes studied.

Primer	Reference	SSR motive	Annealing temp (°C)	Observed alleles	Effective alleles	Shannon’s index	Observed heterozygosity	Expected heterozygosity	Genetic differentiation coefficient	Gene flow
AMPA101	Hagen *et al.* [ 51]	(TC) _11_(AC) _12_	56	5	4.7825	1.5882	0.5119	0.7925	0.1268	1.7221
AMPA119	Hagen *et al.* [ 51]	(TA) _9_	57	7	5.6433	1.8253	0.5714	0.8244	0.1964	1.0230
BPPCT039	Dirlewanger *et al.* [ 50]	(GA) _20_	55	5	4.4628	1.5488	0.4444	0.7775	0.1223	1.7946
pchgms3	Sosinski *et al.* [ 54]	(CT) _19_	57	5	3.5636	1.3522	0.4921	0.7208	0.1726	1.1986
pchgms5	Sosinski *et al.* [ 54]	(CA) _9_(TA) _8_	51	2	1.9027	0.6673	0.5357	0.4754	0.0872	2.6160
ssrPaCITA23	Lopes *et al.* [ 52]	(AC) _2_(AG) _18_	51	6	4.5883	1.6390	0.8651	0.7836	0.0357	6.7527
UDAp-414	Messina *et al.* [ 53]	(AG) _21_	56	4	3.3321	1.2652	0.1944	0.7013	0.3336	0.4995
UDAp-415	Messina *et al.* [ 53]	(GA) _21_	56	4	3.6956	1.3480	0.4683	0.7309	0.1658	1.2577
UDAp-420	Messina *et al.* [ 53]	(CT) _20_	56	5	3.9018	1.3908	0.6429	0.7452	0.1196	1.8403
UDP96-001	Cipriani *et al.* [ 49]	(CA) _17_	57	2	1.8529	0.6529	0.2500	0.4612	0.1871	1.0860
Mean	-	-	-	4.5	3.7726	1.3278	0.4976	0.7013	0.1543	1.3706

**Table S3.** Nei’s (1978) unbiased genetic identity (above diagonal) and genetic distance (below diagonal) among Siberia apricot populations reveled by ISSR markers [62].

POP	LY	ZD	AS	HC	HX	ZY	YG	GL	HY	YQ	HR	MY	CY	KZ	LiY	WC	LH	PQ	NC	LX	KK
**LY**	-	0.9246	0.9248	0.9363	0.9112	0.9125	0.9192	0.9124	0.8673	0.8438	0.8695	0.8911	0.8425	0.8553	0.8775	0.8641	0.8645	0.8543	0.8437	0.8394	0.8399
**ZD**	0.0784	-	0.9505	0.9616	0.9528	0.9509	0.9466	0.9309	0.9161	0.8839	0.8925	0.9205	0.8779	0.8758	0.8888	0.8807	0.8861	0.8807	0.8697	0.8707	0.8688
**AS**	0.0782	0.0507	-	0.9637	0.9426	0.9406	0.9429	0.9239	0.9017	0.8797	0.8999	0.9282	0.8969	0.8903	0.9067	0.8948	0.8865	0.8949	0.8735	0.8799	0.8708
**HC**	0.0658	0.0391	0.037	-	0.9519	0.9467	0.9589	0.9306	0.9089	0.8837	0.8942	0.9317	0.8784	0.879	0.9068	0.8986	0.8997	0.8962	0.8914	0.8769	0.8717
**HX**	0.093	0.0483	0.0591	0.0492	-	0.967	0.9592	0.9324	0.939	0.8795	0.8992	0.9092	0.8613	0.8689	0.8749	0.8798	0.8786	0.8722	0.8815	0.878	0.8831
**ZY**	0.0916	0.0503	0.0612	0.0548	0.0336	-	0.9549	0.9139	0.9186	0.8872	0.8951	0.9167	0.8921	0.8922	0.9032	0.9093	0.8928	0.8806	0.8733	0.8812	0.884
**YG**	0.0843	0.0549	0.0588	0.042	0.0417	0.0461	-	0.9438	0.9293	0.8793	0.896	0.9114	0.8726	0.8659	0.8879	0.9	0.8942	0.885	0.8782	0.8762	0.878
**GL**	0.0917	0.0716	0.0792	0.0719	0.07	0.0901	0.0578	-	0.9406	0.8989	0.8978	0.911	0.8732	0.8875	0.8876	0.8898	0.8924	0.8868	0.8661	0.8805	0.8994
**HY**	0.1424	0.0876	0.1035	0.0955	0.063	0.0849	0.0733	0.0612	-	0.9511	0.9133	0.9248	0.8989	0.9054	0.9022	0.9092	0.9108	0.9178	0.9036	0.9123	0.9232
**YQ**	0.1698	0.1234	0.1282	0.1237	0.1284	0.1197	0.1286	0.1066	0.0502	-	0.9397	0.9441	0.9452	0.956	0.9424	0.9461	0.9341	0.9477	0.9244	0.9386	0.9471
**HR**	0.1398	0.1137	0.1055	0.1119	0.1063	0.1108	0.1098	0.1078	0.0906	0.0622	-	0.9578	0.9216	0.9251	0.9376	0.934	0.9204	0.933	0.9169	0.9339	0.9183
**MY**	0.1153	0.0829	0.0745	0.0708	0.0952	0.087	0.0928	0.0932	0.0781	0.0575	0.0431	-	0.939	0.9334	0.9461	0.9318	0.92	0.9411	0.929	0.9283	0.9145
**CY**	0.1714	0.1302	0.1088	0.1296	0.1493	0.1141	0.1363	0.1356	0.1066	0.0564	0.0817	0.0629	-	0.9837	0.9677	0.9665	0.9272	0.9262	0.895	0.926	0.9187
**KZ**	0.1563	0.1326	0.1161	0.1289	0.1406	0.1141	0.144	0.1194	0.0994	0.045	0.0779	0.0689	0.0164	-	0.968	0.9635	0.9335	0.9338	0.9112	0.9296	0.935
**LiY**	0.1307	0.1178	0.0979	0.0978	0.1336	0.1018	0.1189	0.1192	0.1029	0.0593	0.0645	0.0554	0.0329	0.0325	-	0.9768	0.9427	0.9425	0.9128	0.9342	0.9258
**WC**	0.146	0.127	0.1111	0.1069	0.128	0.0951	0.1053	0.1168	0.0952	0.0554	0.0682	0.0706	0.0341	0.0372	0.0235	-	0.9584	0.9422	0.901	0.9286	0.9377
**LH**	0.1457	0.1209	0.1205	0.1057	0.1295	0.1134	0.1118	0.1139	0.0935	0.0682	0.083	0.0834	0.0755	0.0688	0.059	0.0425	-	0.9587	0.9282	0.9422	0.9448
**PQ**	0.1575	0.127	0.111	0.1096	0.1368	0.1272	0.1221	0.1201	0.0858	0.0537	0.0693	0.0607	0.0767	0.0685	0.0592	0.0595	0.0421	-	0.9688	0.9662	0.9508
**NC**	0.1699	0.1396	0.1352	0.1149	0.1261	0.1354	0.1299	0.1438	0.1013	0.0786	0.0867	0.0737	0.1109	0.0929	0.0912	0.1043	0.0745	0.0317	-	0.9721	0.9388
**LX**	0.1751	0.1385	0.128	0.1313	0.1301	0.1264	0.1322	0.1272	0.0918	0.0633	0.0684	0.0744	0.0769	0.073	0.068	0.0741	0.0595	0.0343	0.0283	-	0.9693
**KK**	0.1744	0.1406	0.1383	0.1373	0.1243	0.1233	0.1301	0.106	0.0799	0.0543	0.0852	0.0894	0.0848	0.0673	0.0771	0.0643	0.0568	0.0504	0.0632	0.0311	-

The “-” means the boundary.

**Table S4.** Nei’s (1978) unbiased genetic identity (above diagonal) and genetic distance (below diagonal) among Siberia apricot populations reveled by SRAP markers [62].

POP	LY	ZD	AS	HC	HX	ZY	YG	GL	HY	YQ	HR	MY	CY	KZ	LiY	WC	LH	PQ	NC	LX	KK
**LY**	-	0.9636	0.9757	0.9729	0.9744	0.9718	0.961	0.9439	0.947	0.9011	0.9272	0.9497	0.9358	0.9238	0.9258	0.9127	0.8983	0.9065	0.9186	0.9033	0.873
**ZD**	0.0371	-	0.974	0.9716	0.9536	0.9688	0.9606	0.9294	0.9374	0.9001	0.9046	0.9227	0.9168	0.8995	0.9044	0.8971	0.8838	0.8919	0.9044	0.8901	0.8613
**AS**	0.0246	0.0263	-	0.981	0.9692	0.9699	0.9622	0.937	0.9487	0.9118	0.9274	0.9419	0.9337	0.9088	0.9123	0.907	0.8865	0.8976	0.9079	0.892	0.8553
**HC**	0.0275	0.0288	0.0192	-	0.9684	0.9701	0.9726	0.9428	0.9477	0.9111	0.9242	0.9388	0.9303	0.9103	0.9132	0.9032	0.8955	0.9043	0.9188	0.8893	0.8664
**HX**	0.0259	0.0475	0.0312	0.0321	-	0.9762	0.9653	0.9527	0.9422	0.9153	0.9363	0.9445	0.932	0.9168	0.9083	0.9024	0.892	0.8908	0.9122	0.8978	0.8708
**ZY**	0.0286	0.0317	0.0306	0.0304	0.0241	-	0.9714	0.951	0.9439	0.9213	0.9295	0.9415	0.9264	0.9212	0.9119	0.9012	0.9008	0.8978	0.911	0.901	0.8791
**YG**	0.0398	0.0402	0.0385	0.0278	0.0354	0.029	-	0.9482	0.9491	0.906	0.9295	0.9427	0.9201	0.9063	0.8994	0.8897	0.8883	0.8931	0.9006	0.893	0.8594
**GL**	0.0577	0.0732	0.065	0.059	0.0485	0.0502	0.0531	-	0.9361	0.9197	0.9323	0.9406	0.9416	0.9234	0.905	0.9046	0.9134	0.8904	0.8958	0.8842	0.8719
**HY**	0.0545	0.0646	0.0526	0.0537	0.0595	0.0578	0.0522	0.066	-	0.946	0.9369	0.9547	0.953	0.9323	0.9196	0.928	0.9407	0.9431	0.9226	0.912	0.8855
**YQ**	0.1042	0.1052	0.0923	0.0931	0.0885	0.082	0.0988	0.0837	0.0555	-	0.9365	0.9345	0.9648	0.9639	0.9406	0.9472	0.9437	0.9275	0.9229	0.931	0.9281
**HR**	0.0755	0.1003	0.0754	0.0789	0.0658	0.0731	0.0731	0.0701	0.0652	0.0656	-	0.9817	0.9609	0.9564	0.9469	0.941	0.938	0.9396	0.9498	0.9395	0.9112
**MY**	0.0517	0.0804	0.0599	0.0632	0.0572	0.0602	0.059	0.0612	0.0464	0.0678	0.0185	-	0.9653	0.9584	0.9541	0.9423	0.9449	0.9627	0.9616	0.9423	0.9099
**CY**	0.0664	0.0869	0.0686	0.0722	0.0705	0.0764	0.0833	0.0602	0.0481	0.0359	0.0398	0.0353	-	0.9859	0.9649	0.9625	0.958	0.9551	0.9521	0.9462	0.9235
**KZ**	0.0792	0.1059	0.0957	0.094	0.0868	0.0821	0.0984	0.0797	0.0701	0.0368	0.0446	0.0425	0.0142	-	0.9702	0.9599	0.9498	0.9515	0.9462	0.9471	0.9325
**LiY**	0.0771	0.1005	0.0918	0.0908	0.0962	0.0922	0.1061	0.0998	0.0838	0.0613	0.0545	0.047	0.0357	0.0302	-	0.9817	0.9482	0.9568	0.9528	0.9547	0.9272
**WC**	0.0914	0.1086	0.0976	0.1018	0.1027	0.104	0.1168	0.1002	0.0747	0.0543	0.0608	0.0595	0.0382	0.0409	0.0184	-	0.9611	0.967	0.9573	0.9559	0.943
**LH**	0.1072	0.1235	0.1205	0.1103	0.1143	0.1045	0.1185	0.0906	0.0611	0.058	0.064	0.0567	0.0429	0.0515	0.0532	0.0397	-	0.9701	0.9469	0.9547	0.9466
**PQ**	0.0982	0.1144	0.108	0.1006	0.1156	0.1079	0.113	0.1161	0.0585	0.0752	0.0623	0.038	0.0459	0.0497	0.0441	0.0336	0.0304	-	0.9718	0.9621	0.9281
**NC**	0.0849	0.1005	0.0966	0.0847	0.0919	0.0932	0.1047	0.11	0.0806	0.0803	0.0515	0.0392	0.0491	0.0553	0.0484	0.0436	0.0545	0.0286	-	0.9777	0.9422
**LX**	0.1017	0.1164	0.1143	0.1173	0.1078	0.1043	0.1132	0.1231	0.0921	0.0715	0.0624	0.0594	0.0553	0.0544	0.0464	0.0451	0.0463	0.0387	0.0225	-	0.9706
**KK**	0.1359	0.1493	0.1563	0.1434	0.1383	0.1289	0.1515	0.1371	0.1216	0.0746	0.093	0.0944	0.0796	0.0699	0.0756	0.0587	0.0549	0.0746	0.0595	0.0298	-

The “-” means the boundary.

**Table S5.** Nei’s (1978) unbiased genetic identity (above diagonal) and genetic distance (below diagonal) among Siberia apricot populations reveled by SSR markers [62].

POP	LY	ZD	AS	HC	HX	ZY	YG	GL	HY	YQ	HR	MY	CY	KZ	LiY	WC	LH	PQ	NC	LX	KK
**LY**	-	0.8003	0.8311	0.7872	0.8099	0.8672	0.809	0.729	0.6243	0.6804	0.6218	0.6774	0.677	0.6046	0.546	0.545	0.6504	0.7714	0.7101	0.7559	0.6019
**ZD**	0.2228	-	0.9443	0.8546	0.8016	0.8244	0.8604	0.8024	0.7017	0.6287	0.6696	0.6399	0.7268	0.6782	0.6801	0.6508	0.656	0.6962	0.5933	0.6535	0.617
**AS**	0.185	0.0573	-	0.8464	0.8379	0.8475	0.9035	0.83	0.7434	0.6438	0.6488	0.7216	0.8105	0.6975	0.6991	0.6934	0.7128	0.7846	0.702	0.6994	0.6562
**HC**	0.2392	0.1571	0.1668	-	0.7722	0.7341	0.7711	0.6664	0.6516	0.6127	0.7047	0.7677	0.7633	0.7042	0.7333	0.6802	0.6064	0.7278	0.6794	0.5961	0.5919
**HX**	0.2108	0.2212	0.1769	0.2585	-	0.827	0.8947	0.7989	0.747	0.682	0.7273	0.6751	0.7476	0.6368	0.5905	0.577	0.762	0.7316	0.6233	0.7329	0.6726
**ZY**	0.1425	0.1931	0.1655	0.3091	0.1899	-	0.8851	0.8579	0.7143	0.7104	0.7548	0.7583	0.7573	0.6788	0.6487	0.6829	0.8021	0.7641	0.7384	0.808	0.7918
**YG**	0.2119	0.1504	0.1015	0.26	0.1112	0.122	-	0.8299	0.8099	0.6677	0.7073	0.7239	0.7122	0.5829	0.6231	0.5759	0.7519	0.7415	0.7034	0.6997	0.696
**GL**	0.3161	0.2201	0.1863	0.4059	0.2245	0.1533	0.1864	-	0.6919	0.7172	0.7896	0.7092	0.7029	0.6491	0.667	0.6641	0.8048	0.7257	0.6277	0.7291	0.7736
**HY**	0.4712	0.3543	0.2965	0.4284	0.2917	0.3365	0.2109	0.3683	-	0.9012	0.7513	0.7189	0.766	0.7634	0.6967	0.6836	0.7802	0.6988	0.6088	0.5735	0.7292
**YQ**	0.3851	0.4641	0.4404	0.4898	0.3827	0.3419	0.404	0.3324	0.1041	-	0.8894	0.7893	0.807	0.8517	0.7448	0.7605	0.8324	0.7818	0.6413	0.6261	0.7811
**HR**	0.4752	0.4011	0.4326	0.35	0.3184	0.2813	0.3463	0.2362	0.2859	0.1172	-	0.9001	0.823	0.8558	0.8292	0.8372	0.8457	0.7507	0.6371	0.6412	0.7849
**MY**	0.3895	0.4465	0.3263	0.2644	0.393	0.2766	0.3231	0.3436	0.33	0.2366	0.1053	-	0.8536	0.8227	0.8858	0.8572	0.8454	0.8471	0.8346	0.7321	0.7997
**CY**	0.3901	0.3191	0.2102	0.2702	0.2909	0.2781	0.3394	0.3525	0.2666	0.2144	0.1948	0.1583	-	0.8832	0.8515	0.8431	0.8401	0.8129	0.7762	0.7455	0.8575
**KZ**	0.5033	0.3883	0.3603	0.3507	0.4514	0.3875	0.5397	0.4322	0.27	0.1605	0.1557	0.1952	0.1242	-	0.9143	0.9286	0.8044	0.773	0.6944	0.695	0.7668
**LiY**	0.6051	0.3855	0.358	0.3102	0.5268	0.4327	0.4731	0.405	0.3615	0.2946	0.1873	0.1212	0.1607	0.0896	-	0.9734	0.8342	0.828	0.7771	0.7336	0.7883
**WC**	0.6069	0.4296	0.3661	0.3853	0.5498	0.3814	0.5519	0.4094	0.3804	0.2738	0.1777	0.1541	0.1706	0.0741	0.027	-	0.8601	0.7937	0.6997	0.6804	0.7524
**LH**	0.4301	0.4215	0.3385	0.5001	0.2718	0.2205	0.2852	0.2172	0.2482	0.1835	0.1676	0.1679	0.1742	0.2176	0.1813	0.1507	-	0.8712	0.8101	0.8705	0.9197
**PQ**	0.2595	0.3621	0.2426	0.3177	0.3125	0.269	0.2991	0.3206	0.3585	0.2462	0.2868	0.166	0.2071	0.2575	0.1888	0.2311	0.1378	-	0.8791	0.882	0.8061
**NC**	0.3423	0.522	0.3539	0.3865	0.4727	0.3032	0.3519	0.4657	0.4963	0.4443	0.4508	0.1808	0.2534	0.3647	0.2522	0.3571	0.2106	0.1288	-	0.8857	0.8414
**LX**	0.2799	0.4254	0.3575	0.5173	0.3108	0.2132	0.3571	0.316	0.556	0.4683	0.4444	0.3119	0.2937	0.3638	0.3097	0.3851	0.1387	0.1256	0.1213	-	0.8669
**KK**	0.5076	0.4829	0.4214	0.5245	0.3967	0.2334	0.3624	0.2568	0.3158	0.2471	0.2422	0.2235	0.1538	0.2656	0.2379	0.2845	0.0837	0.2156	0.1726	0.1428	-

The “-” means the boundary.

Figure S1. Δ*K* values for different numbers of populations assumed (*K*) in the STRUCURE analysis.

Figure S2. Principal component analysis on combined three markers data sets of the entire population.

## Figures and Tables

**Table 1.** Genetic diversity parameters of the *Siberian apricot* populations analyzed.

Population	ISSR			SRAP			SSR	
		
	*h*	*I*	*PPB* (%)	*h*	*I*	*PPB* (%)	*h*	*I*
LY	0.191	0.285	54.4	0.145	0.220	43.3	1.099	0.612
ZD	0.156	0.238	48.5	0.141	0.216	45.0	1.120	0.632
AS	0.200	0.301	59.2	0.165	0.254	55.8	1.231	0.662
HC	0.208	0.310	59.2	0.176	0.271	57.5	1.157	0.631
HX	0.197	0.294	56.3	0.178	0.271	55.0	1.097	0.591
ZY	0.198	0.293	53.4	0.190	0.288	59.2	1.095	0.606
YG	0.156	0.230	42.7	0.158	0.241	49.2	1.134	0.629
GL	0.140	0.212	45.6	0.148	0.228	48.3	1.097	0.596
HY	0.161	0.239	44.7	0.138	0.209	42.5	1.008	0.577
YQ	0.166	0.248	47.6	0.165	0.248	49.2	1.027	0.579
HR	0.204	0.304	58.3	0.213	0.326	69.2	1.106	0.602
MY	0.197	0.291	54.4	0.186	0.283	60.0	1.141	0.633
CY	0.154	0.235	48.5	0.138	0.213	48.3	1.100	0.612
KZ	0.184	0.275	50.5	0.172	0.262	54.2	0.977	0.544
LiY	0.160	0.241	48.6	0.166	0.254	55.0	1.025	0.573
WC	0.161	0.242	47.6	0.149	0.230	50.0	0.945	0.541
LH	0.174	0.259	50.5	0.137	0.210	45.8	1.081	0.592
PQ	0.144	0.221	47.6	0.134	0.213	50.0	1.133	0.621
NC	0.161	0.243	48.5	0.146	0.226	52.5	0.983	0.554
LX	0.161	0.238	43.7	0.132	0.200	40.8	0.891	0.512
KK	0.139	0.211	41.8	0.120	0.183	36.7	0.939	0.534
Mean	0.172	0.258	50.1	0.157	0.240	50.8	1.066	0.592
At the species level	0.248	0.387	91.3	0.218	0.344	82.5	1.639	0.782

*h*, Nei’s gene diversity index; *I*, Shannon’s information index; *PPB*, the percentage of polymorphic bands.

**Table 2.** Analysis of molecular variance (AMOVA) within/among *Siberian apricot* populations.

	ISSR		SRAP		SSR	
			
Source of variance	Variance component	Ratio (%)	Variance component	Ratio (%)	Variance component	Ratio (%)
Among populations	3.098	25.01	3.561	23.84%	1.437	16.65
Within populations	9.289	74.99	11.378	76.16%	7.192	83.35

A *p* value < 0.001 was considered significant.

**Table 3.** Geographical locations of the different *P. sibirica* populations used in this study.

Population code	Seed collection sites	Latitude (°N)	Longitude (°E)	Elevation (m)
LY	Lingyou, Shannxi Province	34°35′	107°46′	1,292
ZD	Zhidan, Shannxi Province	36°48′	108°45′	1,238
AS	Ansai, Shannxi Province	37°04′	109°09′	1,252
HC	Huachi, Gansu Province	36°12′	107°56′	1,244
HX	Huanxian, Gansu Province	36°31′	107°17′	1,190
ZY	Zhenyuan, Gansu Province	35°37′	107°02′	1,281
YG	Yanggao, Shanxi Province	40°07′	113°54′	1,097
GL	Guangling, Shanxi Province	39°49′	114°34′	1,284
HY	Hunyuan, Shanxi Province	39°32′	113°28′	1,442
YQ	Yanqing, Beijing Province	40°26′	116°14′	641
HR	Huairou, Beijing Province	40°36′	116°44′	382
MY	Miyun, Beijing Province	40°31′	117°13′	228
CY	Chaoyang, Liaoning Province	41°42′	120°03′	627
KZ	Kazuo, Liaoning Province	41°01′	119°44′	405
LiY	Lingyuan, Liaoning Province	40°53′	119°12′	560
WC	Weichang, Hebei Province	41°56′	117°44′	1,127
LH	Longhua, Hebei Province	41°15′	117°21′	675
PQ	Pingquan, Hebei Province	41°02′	118°32′	583
NC	Ningcheng, Inner Mongolia	41°45′	119°01′	1,134
LX	Linxi, Inner Mongolia	44°01′	118°20′	1,207
KK	Keshiketeng, Inner Mongolia	43°16′	117°33′	1,221
