# Supplementary figures and images for: Genetic Diversity and Population Structure of Siberian apricot (Prunus sibirica L.) in China
Source: Int J Mol Sci. 2013 Dec 31;15(1):377–400. doi: 10.3390/ijms15010377 (PMC3907815; doi:10.3390/ijms15010377)

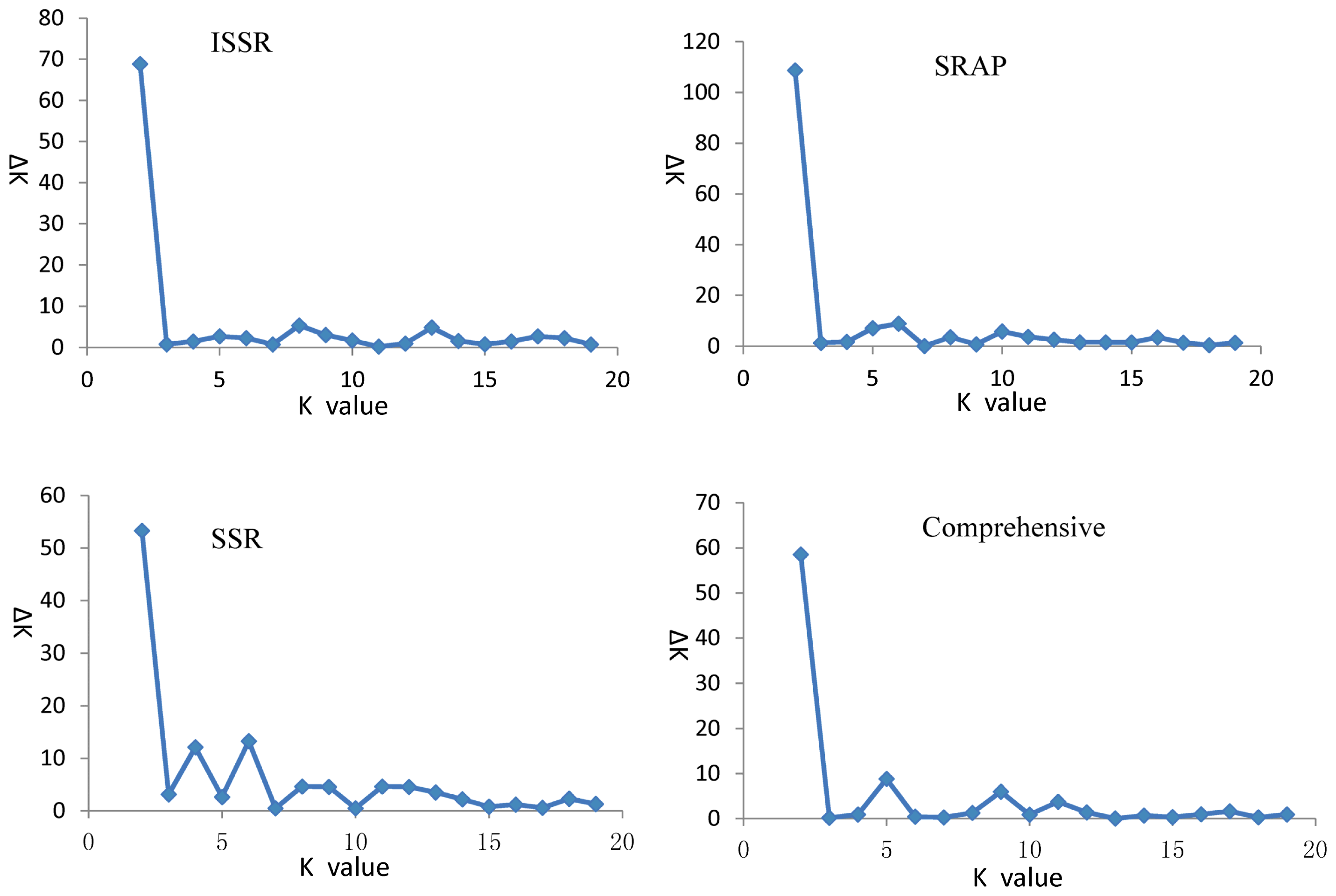

Supplement: Figure S1. — ΔK values for different numbers of populations assumed (K) in the STRUCURE analysis. [file ijms-15-00377s1.tif]

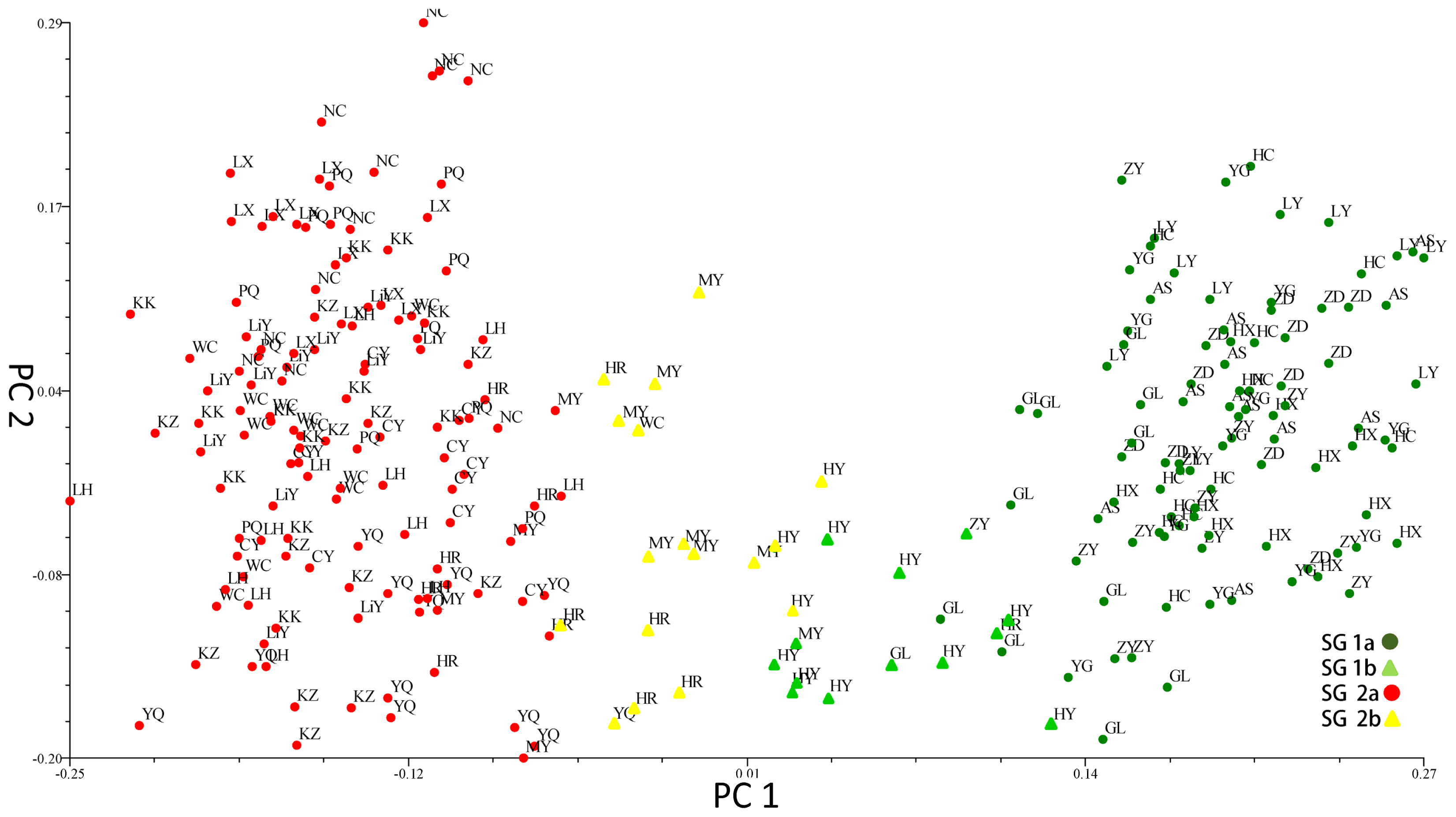

Supplement: Figure S2. — Principal component analysis on combined three markers data sets of the entire population. [file ijms-15-00377s2.tif]
